# The Origin and Evolution of Variable Number Tandem Repeat of *CLEC4M* Gene in the Global Human Population

**DOI:** 10.1371/journal.pone.0030268

**Published:** 2012-01-18

**Authors:** Hui Li, Jia-Xin Wang, Dong-Dong Wu, Hua-Wei Wang, Nelson Leung-Sang Tang, Ya-Ping Zhang

**Affiliations:** 1 Department of Genetics and Developmental Biology, Southeast University School of Medicine and The Key Laboratory of Developmental Genes and Human Disease, Ministry of Education, Southeast University, Nanjing, China; 2 State Key Laboratory of Genetic Resources and Evolution, Kunming Institute of Zoology, Chinese Academy of Sciences, Kunming, China; 3 Laboratory for Conservation and Utilization of Bio-resource & Key Laboratory for Microbial Resources of the Ministery of Education, Yunnan University, Kunming, China; 4 KIZ/CUHK Joint Laboratory of Bioresources and Molecular Research in Common Diseases, Kunming, China; 5 Li Ka Shing Institute of Health Sciences and Department of Chemical Pathology, The Chinese University of Hong Kong, Shatin, Hong Kong, China; 6 Departments of Chemical Pathology, Faculty of Medicine, The Chinese University of Hong Kong, Shatin, Hong Kong, China; 7 Functional Genomics and Biostatistical Computing laboratory, Shenzhen Research Institute, The Chinese University of Hong Kong, Shatin, Hong Kong China; The Centre for Research and Technology, Hellas, Greece

## Abstract

*CLEC4M* is a C-type lectin gene serving as cell adhesion receptor and pathogen recognition receptor. It recognizes several pathogens of important public health concern. In particular, a highly polymorphic variable number tandem repeat (VNTR) at the neck-region of *CLEC4M* had been associated with genetic predisposition to some infectious diseases. To gain insight into the origin and evolution of this VNTR in *CLEC4M*, we studied 21 Africans, 20 Middle Easterns, 35 Europeans, 38 Asians, 13 Oceania, and 18 Americans (a total of 290 chromosomes) from the (Human Genome Diversity Panel) HGDP-CEPH panel; these samples covered most of alleles of this VNTR locus present in human populations. We identified a limited number of haplotypes among the basic repeat subunits that is 69 base pairs in length. Only 8 haplotypes were found. Their sequence identities were determined in the 290 chromosomes. VNTR alleles of different repeat length (from 4 to 9 repeats) were analyzed for composition and orientation of these subunits. Our results showed that the subunit configuration of the same repeat number of VNTR locus from different populations were, in fact, virtually identical. It implies that most of the VNTR alleles existed before dispersion of modern humans outside Africa. Further analyses indicate that the present diversity profile of this locus in worldwide populations is generated from the effect of migration of different tribes and neutral evolution. Our findings do not support the hypothesis that the origin of the VNTR alleles were arisen by independent (separate) mutation events and caused by differential allele advantage and natural selection as suggested by previous report based on SNP data.

## Introduction

Dendritic cell-specific intracellular adhesion molecular-3-grabbing nonintegrin (*CLEC4L*) and L-SIGN also called DC-SIGN related (*CLEC4M*) are C-type lectins involved in both innate and adaptive immunity. They are cell adhesion receptors and pathogen recognition receptors. However, *CLEC4L* (also called CD209) is expressed primarily on phagocytic cells, such as dendritic cells and macrophages, whereas *CLEC4M* (also called CD209L) expression is restricted to endothelial cells in liver and lymph nodes [1], [2]. Both lectins are known to bind multiple pathogens and function as cellular receptors for various viruses, such as HIV-1, Ebola virus, cytomegalovirus, hepatitis C virus, Dengue virus, and SARS-coronavirus [3]–[5]. With regards to *CLEC4M*, it serves as a receptor for a variety of viruses, including HIV-1, hepatitis C, Ebola, and SARS-coronavirus, as well as the parasite Schistosoma mansoni and bacteria *Mycobacterium tuberculosis* [6], [7]. These two lectins are coded by genes that are located within a lectin family genes cluster on chromosome 19p13.2–3. This 26-kb segment is believed to arise as a result of a duplication of an ancestral gene [8]. These two genes in the cluster share a high degree of homology [2]. Both proteins are organized into three domains: (1) an N-terminal cytoplasmic region with a di-leucine motif for internalization followed by a transmembrane domain, (2) a C-terminal extracellular domain with a C-type carbohydrate recognition domain (CRD) involved in pathogen binding, and (3) a neck-region containing variable number of repeats of conserved subunit of 23-amino-acid sequence, that connects the CRD to the transmembrane region.

The neck-region is involved in assembling the lectin into a tetrameric protein conformation on the cell surface, which is believed to be required for efficient recognition of multivalent ligands. And the length variation of this neck-region had been proposed to affect the pathogen-binding properties of the CRD of these proteins [9], [10]. However, this property is controversial [9]. Genetic association studies also showed conflicting results about the associations of length variation of neck-region and several infectious diseases, such as HIV, HCV, and SARS [11]–[19].

*CLEC4M* neck-region exhibits a higher level of heterozygosity due to presence of 4- to 9-repeats in substantial frequencies in the populations. On the other hand, the VNTR in *CLEC4L* is highly conserved (7 repeats is found in >90% in the population). Previous study showed that *CLEC4L* had been under a strong selective constraint, while *CLEC4M* had been shaped by the action of balancing selection, and the VNTR region of these two genes might be the target of such selective pressures [20].

The VNTR repeat length polymorphism in *CLEC4M* has been suggested to be associated with genetic predisposition to infection. We investigated the origin and evolutionary history of this VNTR in human population by clonal sequencing. Samples from the Human Genome Diversity Panel (HGDP)–CEPH panel of different continents were studied. The HGDP-CEPH panel is a resource of 1064 cultured lymphoblastoid cell lines (LCLs) from individuals in 52 different world populations. Information for each LCL is limited to sex, population, and geographic origin of the individual. The panel contains LCLs from populations living on all continents and is collected to provide unlimited supplies of DNA for studies of sequence diversity and history of modern human populations [21].

The sequence identity of each repeat subunit of different VNTR alleles are identified by sequencing after cloning the PCR product and the haplotype structure including identity of each subunit and their orientation of each VNTR (from 4 to 9 repeats) is thus determined.

Our results showed that the haplotype of subunits of the same VNTR (from 4 to 9 repeats) sampled from subjects of different continents were virtually identical, which implied the different VNTR alleles in *CLEC4M* gene had existed for a long time, at least before the date of “out-of-Africa” event. Furthermore, by using the genotype data of VNTR in *CLEC4M* gene and dividing the subjects of the Human Genome Diversity Panel (HGDP)–CEPH panel into 26 populations, we analyzed the correlation between the 25 non-African populations geographic distance to the east Africa and their population genetic distance to the east Africa. We concluded that the present diversity profile of this VNTR in worldwide populations was consistent with demographic migrating of different tribes. No evidence was found to support the notion of independent mutation events due to natural selection.

## Results and Discussion

### Sequence identity of the subunits in VNTR of *CLEC4M* Gene

**Figure 1:**
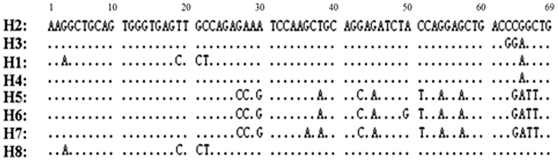
Alignments of all variants of sequences in the 69 base pairs repeat subunit in VNTR. According to their sequence identity, subunits were named as haplotypes H1–H8. Position (base pair) in the subunit is shown on the top. The sequence of H2 is used as the consensus sequence for alignment of other subunit haplotypes. DNA variants are shown as mutation base after alignment. VNTR is assembled from various numbers of these 8 basic haplotype building blocks.

**Figure 2:**
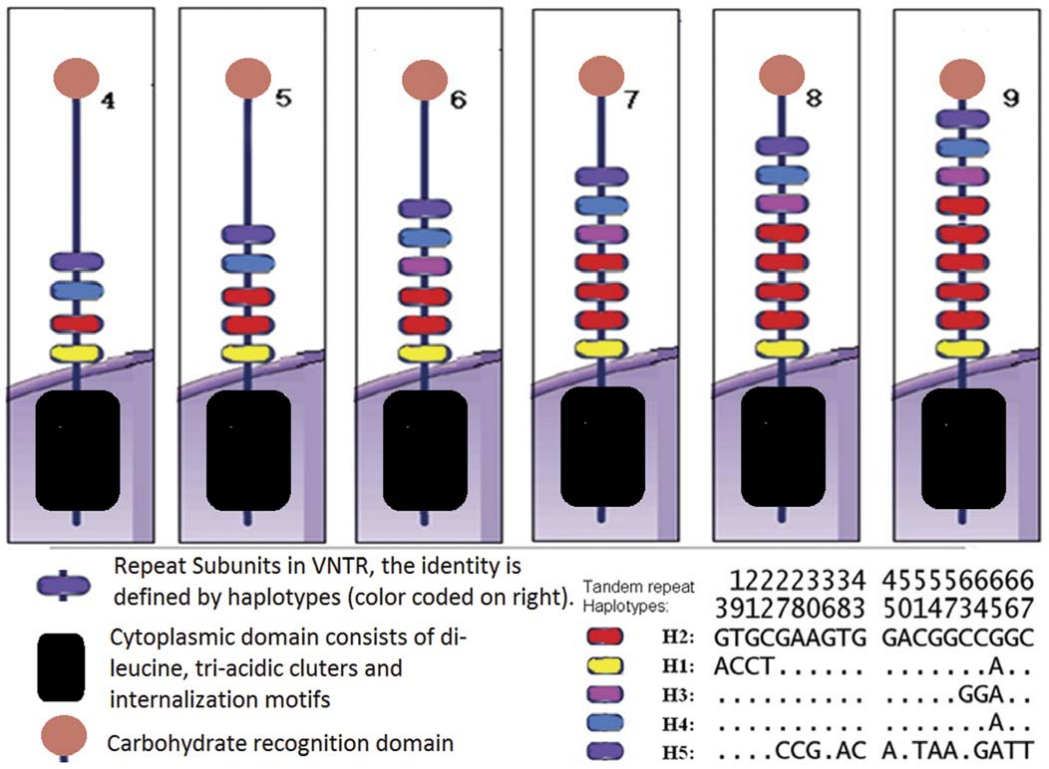
The common composition of subunits of the 4- to 9- repeats at the VNTR locus of *CLEC4M* gene. Different colors are used to represent different repeat (H1–H8 as defined in Figure 1). The lower right panel highlights the sequence alignment against H2.

Work by our team and others showed that the VNTR in *CLEC4M* gene varied in size from 4 to 9 repeats [18]. In this study, we analyzed the sequence identity of each subunit among VNTR of 4 to 9 repeats alleles of 145 samples from different continental populations by clonal sequencing. Figure 1 shows the various haplotypes of each subunit and the prevalent subunit orientation in each VNTR alleles. Only eight different haplotypes account for all sequence variations of all subunits. H2 is the most parsimony one and thus used as the consensus sequence. According to the sequence identity in the 69-bp repeat unit, seven other haplotypes are identified (denoted as H1–H8) (Figure 1). Different numbers of these subunits assemble to form the VNTR alleles of different length. The most prevalent subunit structures of each VNTR are described in Figure 2. The detailed haplotype configurations in VNTR locus for all studied samples are found in Table S1.

### Configuration of Subunit in VNTR alleles

In most VNTR, the N-terminal repeat unit (H1) and C-terminal repeat unit (H4 followed by H5) are highly conserved. Most alleles has H1 and H4 followed by H5 (or their transition variants) in these two locations (Figure 2), which is supportive that these subunits are important for the formation of oligomer of *CLEC4M* [9].

As the 7-repeat allele is the dominant allele in worldwide population, we used the sequencing result of this 7-repeat haplotype as an illustration. One hundred and thirteen alleles of the 7-repeat were sequenced, and 96.5% of them showed identical haplotype and arrangement of subunits. On the direction from the N-terminal to C-terminal, the 7-repeat allele is formed by one H1, three H2, one H3, one H4 and one H5. Thus, it is denoted as H1-H2-H2-H2-H3-H4-H5 in the table of supplementary data (Table S1), and this construction is defined as the common consensus configuration. The consensus configurations of other VNTR alleles are also shown in table 1. For 7-repeat allele, the remainder has one of the three variant configurations which accounted for less than 4%. These variants are due to replacement of one of the H2 subunit by H3 subunit or vice versa. As H2 subunit and H3 subunit differs by three base pairs, it is not caused by a single sporadic event. They represent a signal of independent origin in a small proportion of 7-repeat allele. Therefore, these variants of 7-repeat are classified as major rearrangement in Table 1.

**Table 1:** The proportion of the common consensus configuration, minor variant and major rearrangement in VNTR.

VNTR	4-repeat	5-repeat	6-repeat	7-repeat	8-repeat	9-repeat
common consensus configuration(%)	5/12(41.7%)[H1-H2-H4-H5]	68/71(95.8%)[H1-H2-H2-H4-H5]	35/46(76.1%)[H1-H2-H2-H3-H4-H5]	109/113(96.5%)[H1-H2-H2-H2-H3-H4-H5]	3/3(100%)[H1-H2-H2-H2-H2-H3-H4-H5]	42/45(93.3%)[H1-H2-H2-H2-H2-H2-H3-H4-H5]
minor variant(%)	7/12(58.3%)[**H8**-H2-H4-H5]	3/71(4.2%)[**H8**-H2-H2-H4-H5]	2/46(4.3%)[H1-H2-H2-H3-H4-H6]9/46(19.6%)[H1-H2-H2-H3-H4-H7]	0/113(0%)	0/3(0%)	0/45(0%)
major rearrangement(%)	0/12(0%)	0/71(0%)	0/46(0%)	1/113(0.9%)[H1-H2-H2-H2-**H2**-H4-H5]1/113(0.9%)[H1-H2-H2-**H3**-H2-H4-H5]2/113(1.8%)[H1-H2-H2-**H3**-H3-H4-H5]	0/3(0%)	2/45(4.4%)[H1-H2-H2-H2-**H3**-H2-H3-H4-H5]1/45(2.2%)[H1-H2-H2-**H3**-H2-H2-H3-H4-H5]
total observed number of configurations	2	2	3	4	1	3

Note: minor variant: the difference between two subunits is due to a single base change which also represents a transition change. major rearrangement: change from one subunit haplotype to another with difference in multiple base pairs or a change in the order of subunits. It signifies that the rearrangement cannot be caused by a single sporadic mutation. It may represent an independent origin of the alleles and candidates for natural selection.

When the longer repeat alleles were compared with the shorter one, it is apparent that the expansion of VNTR is due to insertion of H2 subunit (e.g. 8-repeat and 9-repeat). On the other hand, shorter repeat alleles were formed by deletion of H2 subunit. The 7-repeat was the dominant allele and was composed of five haplotype subunits in between the terminal subunits (Figure 2). The 6-repeat, 8-repeat and 9-repeat, had the same haplotype subunit order as the 7-repeat, except for loss or duplication of the subunit H2 (Figure 2).

A dominant consensus configuration can be found in most VNTR alleles. Table 1 shows that only less than 2.5% of the alleles have a major rearrangement. It is found that an independent origin as reflected by prevalence of major rearrangement in a repeat allele was more frequent (6.6%) in the longest allele (9-repeat), suggesting that the longest allele may be found by separate mutagenesis events. On the other hand, all other alleles have only one dominant consensus configuration, together with minor variants of single base transition change which may be due to sporadic transition events. In general, the haplotype subunit and their configuration in each VNTR alleles are common in populations across different continents, and it suggests that they should be originated before the out-of-Africa event, and maintained for a long time.

### Neutral evolution of VNTR in *CLEC4M* Gene

Barreiro et al. have reported that using data of adjacent SNPs of the locus, the *CLEC4M* gene evolved under balancing selection in non-African populations and pointed out that the neck-region was the functional target of such selection [20]. However, we showed that a different mechanism, namely demographic migration might explain equally well the generation of the VNTR diversity based on the sequence subunits within the VNTR repeats.

**Figure 3:**
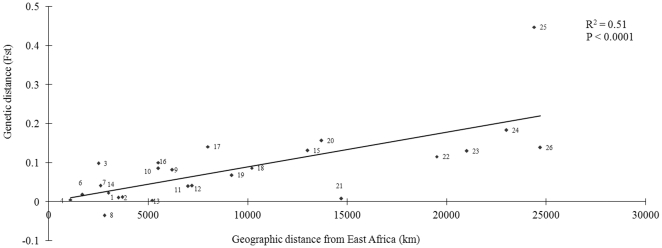
The correlation of the genetic distances (*Fst*) between the 25 non-African populations and the east African population calculated on genotype data of VNTR locus in *CLEC4M* gene and their geographic distances in kilometer (km) from East Africa. The numbers beside the dot represent the 25 populations which were divided and defined according to Barreiro et al 's study [20]. Population numbers were (1) Algerians; (2) Mandenka; (3) Yoruba; (4) Biaka Pygmies; (6) Mbuti Pygmies; (7) San; (8) South African Bantu southeastern/southwestern; (9) French and Basque from France; (10) Italian composite from Bergamo, Tuscany, and Sardinia; (11) Orcadian; (12) Russians; (13) Adygei; (14) Middle Eastern composite sample of Druze, Palestimian, and Bedouin; (15) Yakut; (16) Pakistani composite sample; (17) Chinese composite sample; (18) Japanese; (19) Cambodian; (20) Papuan; (21) Melanesian; (22) Pima; (23) Maya; (24) Piapoco and Curripaco; (25) Surui; and (26) Karitiana.

We divided the individuals of the Human Genome Diversity Panel (HGDP)–CEPH panel into 26 populations according to Barreiro et al.[20]. We used the VNTR locus as a marker to calculate genetic distances between populations. Under the “out-of-Africa” (OFA) model, all modern humans originated from East African [22], it implies that the population from East Africa has the highest genetic diversity, and the genetic distance from non-African to East African declines monotonically with the geographical distance away from East Africa. By defining the northeastern Bantu of Kenya as the Human Original region population, we calculated the genetic distance between the other 25 non-African populations and the original population (East African population). Indeed, the VNTR genetic distances between the East Africa and the 25 different populations significantly correlated with their geographical distance to East Africa (R^2^ = 0.51, P<0.0001 by linear regression) (Figure 3). The data indicated that the present diversity distribution of the VNTR locus in *CLEC4M* gene could be explained by human migration in keeping with the OFA model of human settlement history. The patterns of population variation of VNTR in modern humans could be accounted by neutral evolution, that is, the evolution of VNTR in *CLEC4M* gene was influenced mostly by genetic drift.

In this work, by undertaking a comprehensive clonal sequencing study of the internal structure variation within the observed repeats among different populations at the worldwide scale, we gain insights into the evolutionary mechanism underlying the VNTR of *CLEC4M* gene. The *CLEC4M* gene plays an important role in innate immunity as pathogen receptor and had been studied as susceptibility gene to infectious and generated conflicting results [11]–[13], [15], [16], [18], [19]. As the VNTR is the focus at issue, the study about it is urgent. Understanding of the origin and evolution of the VNTR polymorphism will provide us better insight on whether the VNTR had been a target of natural selection, as an example of the evolution of other immunity related genes. In conclusion, we showed that various VNTR alleles in VNTR locus of *CLEC4M* gene were present before the time of human dispersion out of Africa. Further analysis showed that the present worldwide diversity profile of this VNTR locus could be resulted from demographic migrating of different tribes, and no evidence was found to support the notion of multiple independent mutation events at this VNTR due to natural selection. Future work based on much more comprehensive sequence information from more species, such as chimpanzee, gibbon, bonobo, could further our understanding of evolution of this gene and the effect of different pathogen pressures across the different species of the primates.

## Materials and Methods

### Subjects

A sample of 145 individuals composed of 21 Africans, 20 Middle Easterns, 35 Europeans, 38 Asians, 13 Oceania, and 18 Americans (a total of 290 chromosomes), were chosen from the (Human Genome Diversity Panel) HGDP-CEPH panel. Recruitment of all the individuals met the criteria set by the HGDP Ethics Committee. The ethics of informed consent are specifically addressed by the HGDP's “Model Ethical Protocol for Collecting DNA Samples”, which has been published as [23]. Our present study was performed according to the Declaration of Helsinki. This sample set covered all types of repeats (4- to 9-repeat) in VNTR locus in each continent, based on the data of the *CLEC4M* neck-region variation in the HGDP-CEPH panel including 1064 individuals from 52 worldwide populations (our unpublished data).

### DNA Genotyping and clonal Sequencing

The VNTR polymorphism of the neck-region was genotyped as described in Li et al [18]. The genotype was determined by separating the PCR products in 3% agarose gel with ethidium bromide staining. To validate the genotyping results, 10% of the samples were re-genotyped by duplicated genotyping experiments.

Clonal sequencing of VNTR in *CLEC4M* gene was performed by directly sequencing of cloned PCR products. First, the fragment of exon 4 in *CLEC4M* gene was amplified by the primers, F- CCCTAA GTCAGGAACAATCCGA, R- TCACAGGGGAGGAAACTGAG. PCR was performed in a 50 µl volume with 50 ng genomic DNA as template, 5 µl PCR 10× buffer, 4 µl dNTP mix (2.5 mM), 2 µl primers (10 pM), 1.25 unit of Taq DNA polymerase (Takara) and 38 µl D.W water. Thermal cycling in a Gene Amp 9700 (Perkin Elmer) thermal cycler was performed at 95°C for 5 min, followed by 40 cycles at 95°C for 20 s, 59°C for 30 s, 72°C for 60 s and extension at 72°C for 5 min. The PCR products were purified on spin columns (Watson BioTechnologies Inc., Shanghai) according to manufacturer's protocol. Then, the purified PCR products were cloned into PMD 18-T Vector (Takara, Japan) and transferred into an ultracompetent cell (Takara, Japan). Then, plasmids carrying a PCR fragment were extracted and sequenced in both directions directly by primers, SF-AGGAACAATCCGAGCAAGACG and SR-AGAGACCATCTCAGGCCCAAG, with an ABI 3730 automatic sequencer. For each PCR product, at least 5 clones were chosen at random and sequenced in order to reduce artifactual error leading to single nucleotide variants and artificial recombination due to PCR. The sequencing results were processed by DNASTAR software (DNASTAR) and manual rectification.

### Sequence identity (Haplotype) of VNTR Subunits

As the VNTR is composed of a variable number of a basic subunit of 69 base pairs, we analyzed all the sequence variants within each of the subunit. Then, all sequence reads of the 69-bp subunit of VNTR were aligned using the Clustal W program [24]. We only observed 8 different sequence haplotypes (Figure 1). According to the sequence variation in the 69-bp fragment, all the subunits could be unique named as one of the 8 different haplotypes.

### Composition of subunits within various VNTR alleles

The clonal sequence of the 290 chromosomes of the worldwide population sample then called according to the subunit haplotype (H1 to H8). For example, the common 7-repeat VNTR allele is formed by one H1, three H2, one each of H3, H4, and H5 in the order of H1-H2-H2-H2-H3-H4-H5 (Figure 2 and Table 1).

### Calculating the genetic distances between populations

The genotype data of VNTR locus in *CLEC4M* gene were used to calculate the genetic distance between populations. First, the samples of the Human Genome Diversity Panel (HGDP)–CEPH panel were divided into 26 populations as used in the study of Barreiro et al [20]. Then, the population from northeastern Bantu of Kenya was defined as the Human Original region population. Genetic distances between populations (*Fst*) (the other 25 non-African populations and the defined original region's population) were estimated using Arlequin package [25]. Correlation analysis between the graphical distance and genetic population distance was carried out using Pearson bivariate correction analysis (SPSS for Windows; 13.0).

## Supporting Information

Table S1 Detailed haplotype configurations in VNTR locus for all studied samples.
(XLS)
